# A higher spectral range of beetle bioluminescence with infraluciferin

**DOI:** 10.3389/fbioe.2022.897272

**Published:** 2022-08-26

**Authors:** Amit P. Jathoul, Bruce R. Branchini, James C. Anderson, James A. H. Murray

**Affiliations:** ^1^ School of Biosciences, University of Cardiff, Cardiff, United Kingdom; ^2^ Bioflares Ltd., Trowbridge, Wiltshire, United Kingdom; ^3^ Hale Laboratory, Connecticut College, New London, CT, United States; ^4^ Department of Chemistry, University College London, London, United Kingdom

**Keywords:** bioluminescence, green, near-infrared, multicolor, infraluciferin, luciferase, spectral range

## Abstract

Coleopteran bioluminescence is unique in that beetle luciferases emit colors ranging between green (ca.550 nm) and red (ca.600 nm), including intermediate colors such as yellow and orange, allowing up to 3 simultaneous parameters to be resolved *in vitro* with natural luciferin (*D*-LH_2_). Here, we report a more than doubling of the maximum bioluminescence wavelength range using a single synthetic substrate, infraluciferin (iLH_2_). We report that different luciferases can emit colors ranging from visible green to near-infrared (nIR) with iLH_2,_ including in human cells. iLH_2_ was designed for dual color far-red to nIR bioluminescence imaging (BLI) in small animals and has been utilized in different mouse models of cancer (including a metastatic hepatic model showing detailed hepatic morphology) and for robust dual parameter imaging *in vivo* (including in systemic hematological models). Here, we report the properties of different enzymes with iLH_2_: Lampyrid wild-type (WT) *Photinus pyralis* (*Ppy*) firefly luciferase, *Ppy*-based derivatives previously engineered to be thermostable with *D*-LH_2_, and also color-shifted Elaterid-based enzymes: blue-shifted *Pyrearinus termitilluminans* derivative Eluc (reported D-LH_2_ λmax = 538 nm) and red-shifted *Pyrophorus plagiopthalamus* derivative click beetle red (CBR) luciferase (*D*-LH_2_ λmax = 618 nm). As purified enzyme, in bacteria or in human cells, Eluc emitted green light (λmax = 536 nm) with *DL*-iLH_2_ whereas *Ppy* Fluc (λmax = 689 nm), x2 Fluc (λmax = 704 nm), x5 Fluc (λmax = 694 nm), x11 Fluc (λmax = 694 nm) and CBR (λmax = 721 nm) produced far-red to nIR peak wavelengths. Therefore, with iLH_2,_ enzyme λmaxes can be separated by ca.185nm, giving almost non-overlapping spectra. This is the first report of single-substrate bioluminescence color emission ranging from visible green to nIR in cells and may help shed light on the color tuning mechanism of beetle luciferases. We also report on the reason for the improvement in activity of x11 Fluc with iLH_2_ and engineer an improved infraluciferase (iluc) based on this mutant.

## 1 Introduction

**FIGURE 1:**
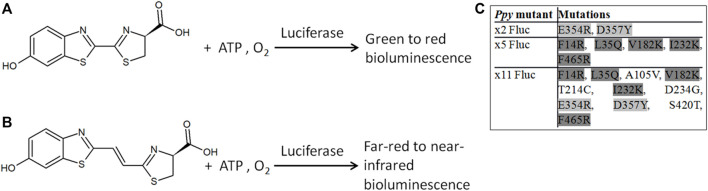
Bioluminescence reactions of beetle luciferases. Bioluminescence with *D*-LH_2_ **(A)** and *D*-iLH_2_ **(B)** (4S)-2-[(E)-2-(6-hydroxy-1,3-benzothiazol-2-yl)ethenyl]-4,5-dihydro-1,3-thiazole-4-carboxylic acid; ATP: adenosine triphosphate. **(C)** Inset table of different subsets of mutations in thermostable mutants of *Ppy* Fluc: x2 Fluc (Willey et al., 2001), x5 Fluc (Law et al., 2006), and x11 Fluc (Jathoul et al., 2012a).

Beetle luciferases catalyze a reaction of beetle *D*-luciferin (*D*-LH_2_), adenosine triphosphate (ATP), and oxygen to produce bright genetically encodable light of colors ranging from green to red (Viviani and Ohmiya, 2000; Nakajima et al., 2005). This widely studied reaction has numerous applications, including being used for molecular diagnostics (Kiddle et al., 2012) and BLI in biomedicine (Badr and Tannous, 2011), helping unravel mammalian molecular and cellular mechanisms or responses to therapies. However, applications in mammalian tissues are limited by the presence of oxy- and deoxy-hemoglobin (HbO_2_ and Hb), which absorb light at <600 nm wavelengths (Liang et al., 2012)^,^ (Li et al., 2009), complicating signal rendering and quantification of BLI and dual parameter BLI *in vivo* (Rice et al., 2001; Zhao et al., 2005; Rice and Contag, 2009). As mammalian tissues are relatively transparent to wavelengths of 650–1350 nm (the bio-optical window), to overcome these challenges, we previously described infraluciferin (iLH_2_), a red-shifted analog of *D*-LH_2_ (Figure 1) that can produce different far-red to nIR colors with different luciferases in the bio-optical window of mammalian tissues (Jathoul et al., 2014; Anderson et al., 2017; Stowe et al., 2019). In other words, wavelength shifts that were normally only observed with *D*-LH_2_ were observed at longer wavelengths with iLH_2_, demonstrating color tuning with a red-shifted analog. BLI in mice using *D*-iLH_2_ methyl (Me) ester (or even *DL*-iLH_2_ Me ester) with wild-type (WT) *Photinus pyralis* (*Ppy*) firefly luciferase (Fluc) and its thermostable/color derivatives previously allowed nIR BLI of detailed disease morphologies in small animal models of cancer (Jathoul et al., 2014) and allowed robust dual color imaging of T-cell effectors and cancer targets in a Chimeric Antigen Receptor (CAR) T-cell cancer therapy model *in vivo* (Stowe et al., 2019), showing its advantages for BLI compared to *D*-LH_2._ However, pure WT *Ppy* Fluc enzyme produced much lower specific activity with *DL*-iLH_2_ than with *D*-LH_2_, by over 3-orders of magnitude at pH 7.8 (Anderson et al., 2019). x11 Fluc (Jathoul et al., 2012a) was found to be our most active mutant, approximately 7-fold brighter, and contained subsets of mutations previously engineered in Murray Lab (Figure 1). As there is a significant reduction of *in vivo* attenuation by Hb/HbO_2_ using iLH_2_, x11 Fluc and x11 Fluc color derivatives (Jathoul et al., 2012b) have proven efficient, and the single-substrate dual-parameter *in vivo* BLI approach made possible by iLH_2_ is attractive owing to homogeneous *in vivo* pharmacokinetics and dynamics of the single substrate in simultaneous or consecutive acquisitions.

Here, we report some basic properties of beetle luciferases with *DL*-iLH_2_, such as specific activities, kinetics, and pH dependence of activities and colors. We established some foundational aspects of *Ppy*-based Fluc activities and developed brighter infraluciferases (ilucs) based on the x11 Fluc scaffold. To better understand color shifting potential with *DL*-iLH_2_, we also examined two of the most extremely color-shifted luciferases: Eluc, derived from *Pyrearinus termitiluminans* luciferase, is one of the most blue-shifted enzymes reported with *D*-LH_2_ (λmax = 538 nm) (Neto et al., 2009)^,^ (Nakajima et al., 2010) and click beetle red (CBR) luciferase, from *Pyrophorus plagiopthalamus* luciferase, one of the most red-shifted (*D*-LH_2_ λmax = 618) (Miloud et al., 2007), close to the most red-shifted enzyme with *D*-LH_2_ from *Phrixotrix hirtus* (PxRE, *D*-LH_2_ λmax = 623) (Bevilaqua et al., 2019).

## 2 Results and discussion

### 2.1 Bioluminescence of beetle luciferase enzymes with luciferin and infraluciferin

#### 2.1.1 Basic bioluminescence properties of beetle luciferases with infraluciferin

To test conditions in human cells appropriately, WT Fluc, x2 Fluc, x5 Fluc, x11 Fluc, Eluc, and CBR enzymes were purified to measure their basic properties with substrates. We utilized *DL*-iLH_2_ free acid for enzyme work and its carboxy-methyl ester for cell work. The use of the racemic mix was for ease of synthesis, as reported for the development of other red-shifted luciferin analogs (Miura et al., 2013). Similar to that case, we expected that light yields could be in the order of 2–3-times lower than with an enantiopure *D*-iLH_2_. Flash kinetics of enzymes were similar with both *D*-LH_2_ and *DL*-iLH_2_ (Supplementary Figure S1A–C), suggesting that adenylation of *DL*-iLH_2_ proceeds effectively, but oxidation is less efficient. We could not measure Km for *DL*-iLH_2_ due to the presence of the inhibitory *L*-form. Kms for ATP in the presence of *DL*-iLH_2_ (ATP_(iLH2)_) are significant to imaging in cells (Branchini et al., 2015) and were found to be in the range of 200–300 μM for all enzymes (Supplementary Table S1). pH dependence of activity measurements with *DL*-iLH_2_ showed that Eluc, WT *Ppy*, x2, and x5 Flucs had optimal activity in the region of pH 6.6, while x11 Fluc was at pH 7.8, and CBR had a lower pH optimum of pH 5.6 (Supplementary Figure S2).

#### 2.1.2 Activity of beetle luciferases and thermostable mutants with luciferin and infraluciferin

##### 2.1.2.1 Enzyme-specific activities at physiological pH, effect of substrate concentration, coenzyme A and hemoglobin attenuation on specific activity

**FIGURE 2:**
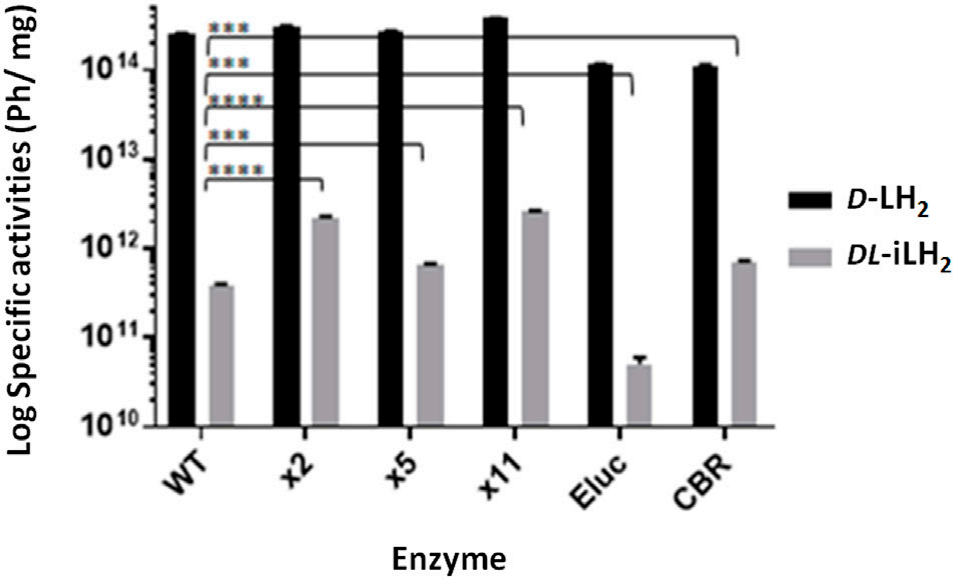
Specific activities of WT *Ppy* Fluc, thermostable *Ppy*-based Fluc mutants, Eluc, and CBR with *DL*-iLH_2_. Specific activity of 0.16 μM enzymes at pH 7.3 in PEM buffer, 200 μM *D*-LH_2_ or *DL*-iLH_2_ and 2 mM ATP, and emission was captured for 3 min through the open filter in the PIO. *p*-values for *t*-test of activity with *DL*-iLH_2_ between WT Fluc and x2 (>0.0001), x5 (0.0003), x11 (>0.0001), Eluc (0.0001), and CBR (0.0002).

Since *DL*-iLH_2_ is for use in cells and animals, we compared the specific activities of pure enzymes at close to physiological pH (7.3) (Figure 2). With 200 μM *DL*-iLH_2_ at pH 7.3, Eluc, WT Fluc, x5 Fluc, and CBR produced 0.02, 0.15, 0.26, and 0.28% of the specific activity of WT Fluc with *D*-LH_2_. In comparison, x2 and x11 Flucs produced 6–7-fold enhanced activity of 0.88 and 1.04%, indicating mutations E354R and/or D357Y are responsible for enhancement. Overall, x11 Fluc was the most active enzyme with *DL*-iLH_2_, maintaining linear emission kinetics and also 5-times higher activity when expressed in *E. coli* (Supplementary Figure S3). Reactions of x2, x5 Flucs, and Eluc were inhibited by increasing concentrations of substrate, possibly due to *L-*iLH_2_ (da Silva and da Silva, 2011). At lower concentrations of *DL*-iLH_2_ (1.5–15 μM), the activity of Eluc was markedly increased to the level of x11 Fluc (Supplementary Figure S4A). Coenzyme A (CoA) improves the activity of Fluc with *D*-LH_2_ (Fraga et al., 2005) and, in an analogous fashion, CoA increased the maximum level of emission 2–5-fold and reduced signal decay of enzymes with *DL*-iLH_2_, with the exception of Eluc, with which CoA reduced activity (Supplementary Figure S4A and B). To account for signal augmentation *in vivo*, we simulated the effect of Hb attenuation with a 1 cm thick agarose phantom containing 50% whole equine blood (Hb concentration estimated by spectrometry to be 0.55 mM) (Supplementary Figure S4C). Through blood, the integrated light emission from WT, x2, x5, or x11 Flucs was attenuated more than 100-fold with *D*-LH_2_ but less than 10-fold with *DL*-iLH_2_.

#### 2.1.2 An expanded spectral range of beetle bioluminescence with infraluciferin

**FIGURE 3:**
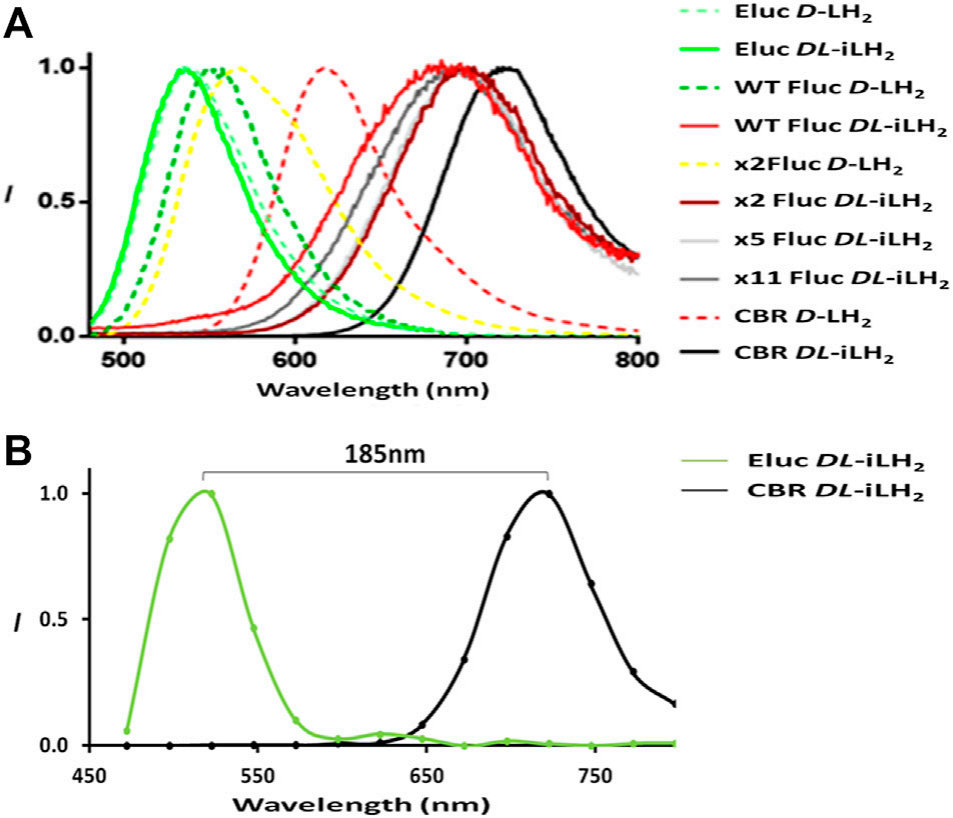
Expanded bioluminescence spectral range with infraluciferin. **(A)** Bioluminescence spectra of different luciferases with *D*-LH_2_ and *DL*-iLH_2_: 5 μM enzymes were assayed with 200 μM luciferins and 2 mM ATP, and light was captured using the Clariostar instrument. For ease of visualization, x5 and x11 Fluc spectra with *D*-LH_2_ are omitted but are near identical to WT Fluc (Reference, Table 1). **(B)** Illustration of spectral separation between Eluc and CBR with *DL*-iLH_2_ measured in the PIO with 0.5 μM enzymes (details as in Supplementary Figure S5).

To examine the emission colors of enzymes with *DL*-iLH_2_, bioluminescence spectra (Figure 3A) were acquired using a Clariostar multimeter (Clariostar, BMG Labtech, Ortenburg, Germany) fitted with a detector module with improved sensitivity to nIR wavelengths and in the PhotonIMAGER Optima (PIO, Biospace Labs, Paris, France) small animal imager (Supplementary Figure S5). Bioluminescence spectra λmax values for WT, x2, x5, and x11 Flucs with *DL*-iLH_2_ were 685, 704, 694, and 694 nm, respectively (Table 1), which 131, 138, 137 and 137 nm shifted compared to their λmaxes with *D*-LH_2_. Full width half maximum (FWHM) values of most enzymes were larger with *DL*-iLH_2_ than with *D*-LH_2_, indicating room for improvement for spectral specificities. Remarkably the emission color of Eluc with *DL*-iLH_2_ was green (from *E. coli* or as purified protein, λmax = 536 nm), and CBR was nIR (λmax = 721 nm), with similar or narrower spectra than with *D*-LH_2_. These click beetle enzymes are extremely blue- and red-shifted and pH-independent in terms of color with *D*-LH_2_ (Viviani, 2002). However, with *DL*-iLH_2_, they produced almost mutually exclusively spectra with a 185 nm peak separation between them (Figure 3B). Therefore, bioluminescence with *DL*-iLH_2_ can range in emission color from the visible to the nIR. This effect has the potential to benefit academia in the future and to multicolor BLI in the absence of Hb. Eluc produced a much smaller secondary nIR peak (ca.700 nm), giving an overall 7.5% overlap between normalized spectra of Eluc and CBR with *DL*-iLH_2_ measured in the PIO, as opposed to 28% with *D*-LH_2_, which was previously one of the largest spectral separations achievable with *D*-LH_2_. Green emission was recently reported from enzymes CBG99 and CBG99opt (λmax = 545 nm) with near-infrared emitting naphthyl amino-luciferin (NH2-NpLH2) (Zambito et al., 2020); however, the supplementary supporting bioluminescence spectra provided for that study show that these enzymes are red for CBG99 (λmax = ca.600 nm) and far-red for CBG99opt (λmax = ca.650 nm) with NH2-NpLH2 (Zambito et al., 2020) in human embryonic kidney (HEK) cells. pH dependence of bioluminescence spectra of *Ppy*-based Flucs with *DL*-iLH_2_ mirrored the classical effects typically obtained with Flucs and *D*-LH_2_ (Supplementary Figure S6, S7): WT Fluc displayed a classic bathochromic shift (Tisi et al., 2002) at lower pH values with *DL*-iLH_2_; Eluc had pH-independent color with *DL*-iLH_2_; CBR displayed a small reciprocal hypsochromic shift at low pH.

**TABLE 1:** Bioluminescence spectral λmax and full-width half maxima. Data were acquired using the Clariostar instrument. Experimental details are as shown in Figure 3A.

Enzyme	Substrate			
	*D*-LH_2_		*DL*-iLH_2_	
	λmax (nm)	FWHM (nm)	λmax (nm)	FWHM (nm)
Eluc	536	71	536	67
WT Fluc	554	68	685	121
x2 Fluc	566	92	704	107
x5 Fluc	557	75	694	104
x11 Fluc	557	75	694	115
CBR	618	74	721	88

Despite Eluc having a low activity with *DL*-iLH_2_, we were confident that this was due to a low rate of oxidation and that the effect was not due to the chemiluminescence of free infraluciferyl adenylate initiated by Eluc. The spectral shape of the green emission was very similar to the narrow spectrum of Eluc with *D*-LH_2_. The activity of Eluc with *DL*-iLH_2_ was dependent on enzyme concentration, though the green spectrum did not vary with enzyme concentration (Figure 3). Chemiluminescence of *DL*-iLH_2_ Me ester initiated using 1M potassium tert-butoxide (t-BuOK) (Miura et al., 2013) was seen to emit broadly in the visible between green and red depending on the buffer, and its shape differed entirely from that of Eluc (Supplementary Figure S8). This result has ramifications for the color tuning mechanism of beetle luciferases (Branchini et al., 2017) (Supplementary Figure S9).

### 2.2 Engineering brighter enzymes with infraluciferin

#### 2.2.1 WT *Ppy* Fluc mutation E354R improves activity with *DL*-iLH_2_ and D357Y red-shifts emission

**FIGURE 4:**
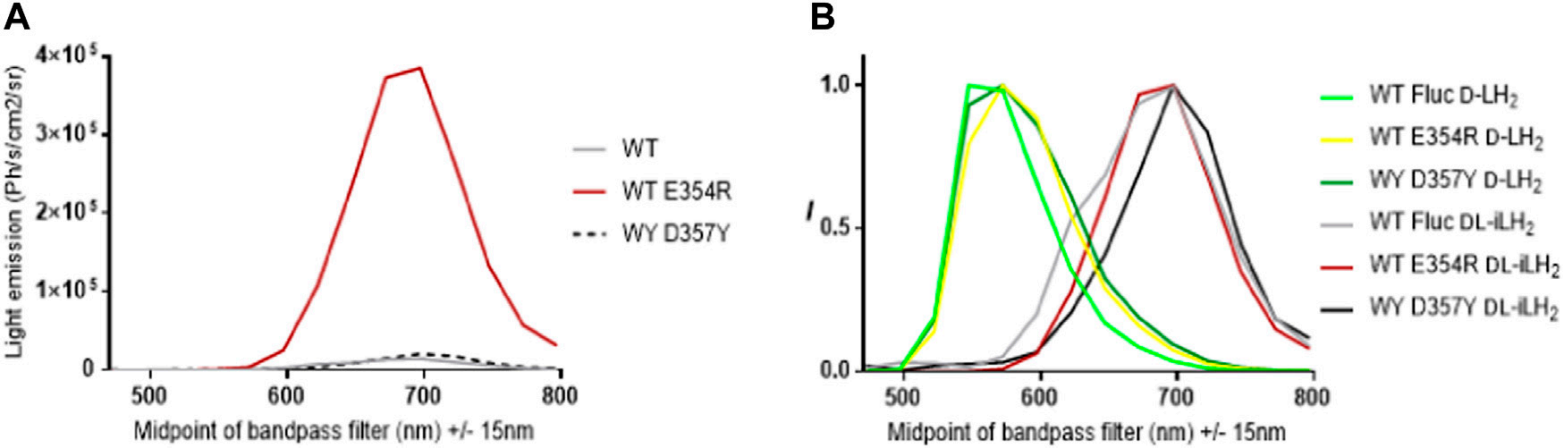
Effect of Fluc mutations E354R and D357Y on activity and color with *DL*-iLH_2_. **(A)** Quantitative bioluminescence spectra showing that E354R leads to significant improvement in activity with *DL*-iLH_2_. **(B)** Normalized bioluminescence spectra of WT *Ppy* Fluc, E354R, and D357Y with *D*-LH_2_ and *DL*-iLH_2_.

To examine the reasons for the relatively higher activities of x2 and x11 Flucs with *DL*-iLH_2_ over WT *Ppy* Fluc, the mutations E354R and D357Y, located in a solvent-exposed omega-loop (Ω-loop) (Halliwell et al., 2018), (Conti et al., 1996) were individually constructed in WT Fluc. Mutations (Willey et al., 2001), insertions (Tafreshi et al., 2007), and deletions (Halliwell et al., 2018) in the *Ω*-loop affect properties such as thermostability and color with *D*-LH_2_ by altering H-bond networks which link adjacent surface loops to enclose the active site in the region that coordinates the 6-hydroxyl of *D*-LH_2_. Deletions in the *Ω*-loop can also affect substrate specificity with *DL*-iLH_2_ (Halliwell et al., 2018). In the *Ppy* Fluc structure with 5′-O-[N-(dehydroluciferyl)-sulfamoyl] adenosine (DLSA) bound (4G36.pdb) (Sundlov et al., 2012), E354 H-bonds to H310, and E311 H-bonds to nearby loop residue R337. This network has been implicated in the stabilization of the hydrophobic active site and providing a counterion for LO phenolate (Viviani et al., 2016). We found that E354R alone in WT Fluc was sufficient to cause an average 3.3-fold improvement in activity with *DL*-iLH_2_ through an open filter on the PIO (Figure 4). Mutation D357Y did not affect activity but red-shifted emission color and double mutant x2 Fluc displayed both effects of improved activity and red-shift. No further improvement was found by screening random mutations at both positions in WT Fluc, and the addition of E354R/D357Y to x5 Fluc only marginally improved activity. Some hints to a mechanism were gained by *in silico* docking (Goodsell and Olson, 1990), suggesting that improved iLH_2_ coordination enhances the light yield of WT Fluc E354R (Berraud-Pache and Naviet, 2016) (Reference Supplementary Figure S10 and S11).

#### 2.2.2 x11 Fluc R354H/Y357A displays enhanced activity specifically with *DL*-iLH_2_

**FIGURE 5:**
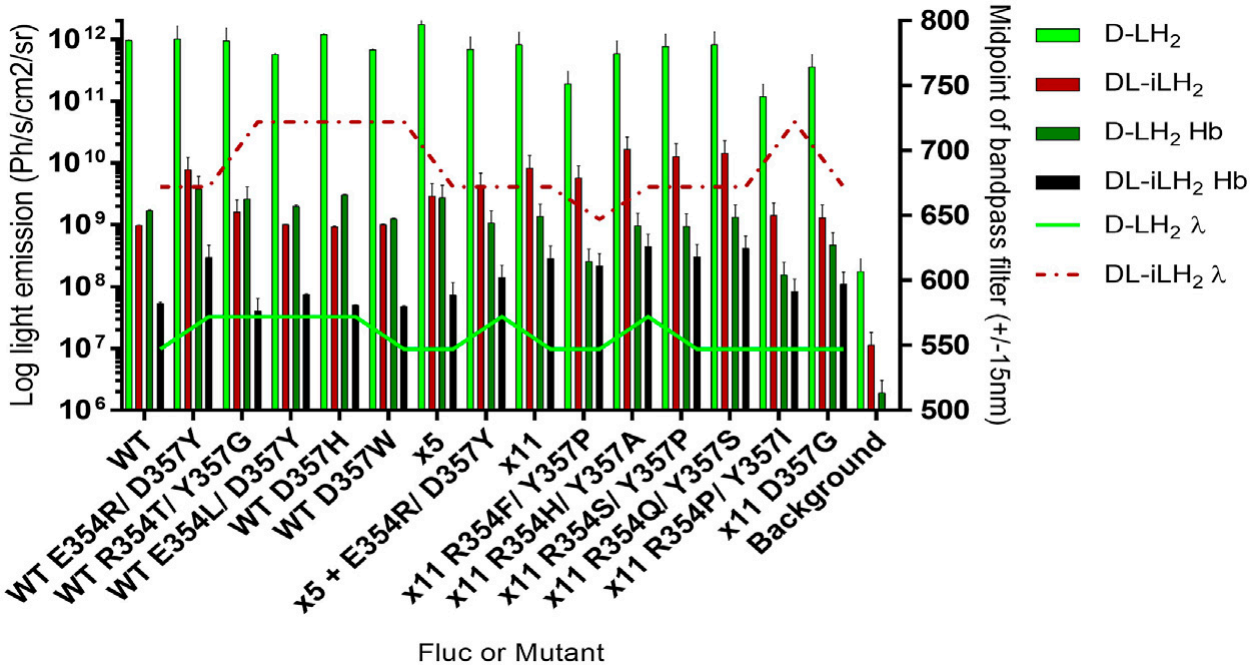
Activity of purified Flucs and mutants with both substrates. Specific activity and emission peak wavelengths of selected R354 and/or D357 mutants of WT, x5, and x11 Flucs. 150μM *D*-LH_2_ or 15 μM *DL*-iLH_2_ and 2 mM ATP were used to saturate 0.167 and 0.0167 μM Flucs, respectively, and light emission was captured using the PIO. The assay was then repeated with the blood phantom.

Since the activity of x11 Fluc with *DL*-iLH_2_ was affected by the conformation of the *Ω*-loop, we randomly mutated both positions singly and together in x11 Fluc and improvements in activity were observed in double mutant screens (Figure 5). Basic and polar residues enhanced activity at position 354, but this depended on the identity of residue 357, at which less polarity appeared favorable. We isolated significantly brighter x11 Fluc mutants, E354H/D357A, E354Q/D357S, and E354S/D357P, with 3-, 2-, and 2.5-fold higher activities than x11 Fluc, respectively. The improved activity of mutant x11 Fluc R354H/Y357A was confirmed separately in the laboratory of the second co-author. However, it was less active than x11 Fluc with *D*-LH_2_, so we termed this mutant infraluciferase 1 (iluc1). Pure iluc1 proved to be up to 17-fold brighter than WT Fluc with *DL*-iLH_2_ and with a small blue-shift in emission compared to WT Fluc and x11 Fluc (Supplementary Figure S12A–C). The pH dependence of specific activity showed that iluc1 had optimum pH at 7.4, compared to 7.8 for x11 Fluc, but the pH affected the emission color of iluc1 more than x11 Fluc, and it displayed a slightly larger bathochromic shift at low pH and reciprocal hypsochromic shift at higher pH with diminution of activity at pH 9.8 (Supplementary Figure S12D–F). The kinetics of iluc1 showed a slightly higher decay than x11 Fluc (Supplementary Figure S12G). Iluc1 proved to be 20-fold brighter than WT Fluc with *DL*-iLH_2_ in *E. coli* BL21 cells (Supplementary Figure S12H).

### 2.3 Properties of iluc1 and color-shifted iluc1 derivatives in human embryonic kidney 293 cells

**FIGURE 6:**
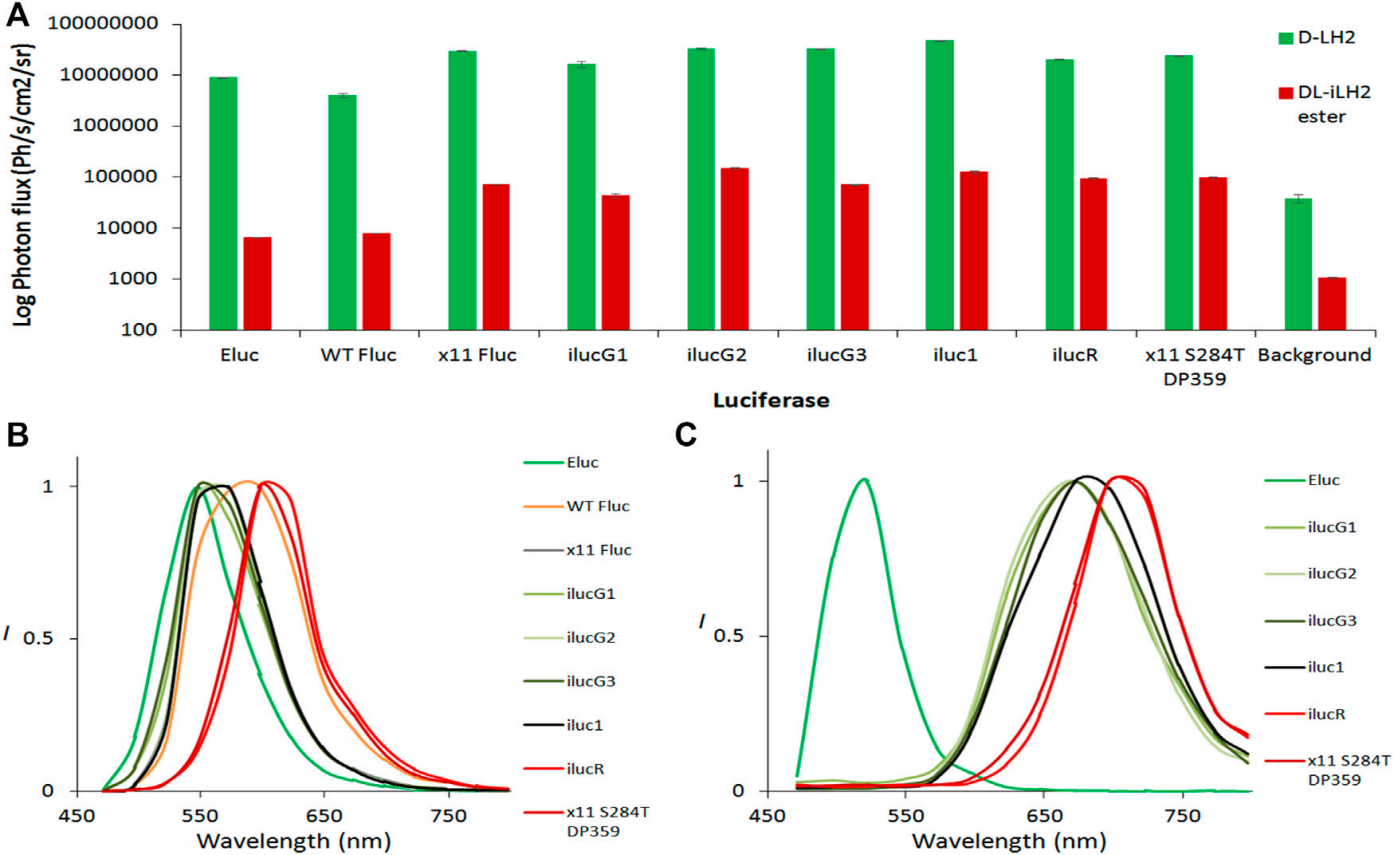
Bioluminescence yields and spectra from transfected HEK 293 cells. **(A)** EGFP normalized light yields from whole HEK cells. 1mM *D*-LH_2_ K^+^ salt or *DL*-iLH_2_ Me ester was added to initiate luminescence, and emission was captured over 5 min at 30°C in the PIO (Biospace Labs, Paris, France). Emission with both substrates was observed to take approximately 1 min to reach a plateau, and light yields given were from 1 min after addition of substrates. Background signals were obtained from triplicate ROIs placed in areas of images containing no wells. **(B)** Normalized bioluminescence spectra obtained from whole transfected HEK cells expressing different variants with **(B)** *D*-LH_2_ and **(C)** *DL*-iLH_2_ Me ester imaged in the PIO.

Human codon-optimized Eluc, WT Fluc, x11 Fluc, iluc1, and CBR were purchased as gblocks (IDT DNA, IA, United States) and cloned using AfeI/XbaI into a lentiviral vector pCCL (Dull et al., 1998) co-expressing EGFP downstream of an internal ribosome entry site (IRES). Vectors encoding different mutants were transfected into human embryonic kidney (HEK) cells and, after 48 h, were imaged for EGFP, followed by BLI by addition of either 1 mM *D*-LH_2_ potassium salt (Regis Tech, IL, United States) or 1 mM *DL*-iLH_2_ Me ester both in phosphate-buffered saline (PBS) onto whole cells with culture medium (containing phenol red) removed. BLI signals were EGFP normalized to account for transfection efficiencies, and no bioluminescence was detected from any non-transfected control cells with either substrate. Eluc and WT *Ppy* Fluc gave 0.17 and 0.2% total integrated light emission with *DL*-iLH_2_ compared to WT Fluc with *D*-LH_2_, whereas with x11 Fluc and iluc1 gave 1.8 and 3.1%, respectively (Figure 6A and Supplementary Figure S13). Eluc activity was weak and did not improve at lower concentrations (100 μM) of *DL*-iLH_2_ ester. Though the green peak was stable in HEK cells, the secondary unstable nIR peak (peaking in the 697 nm +/− 15 nm filter) was observed in some measurements. Iluc1 dual color mutants were constructed by the introduction of mutations V241I, G246A, and F250S to blue-shift and S284T to red-shift spectra (Branchini et al., 2007). x11 Fluc is relatively refractory to classic red-shifting mutation S284T, requiring the additional substitution R354I (“x11 red2”, or “FLuc_red”) (Jathoul et al., 2012b; Stowe et al., 2019) to stabilize red-shift (Willey et al., 2001) for dual imaging with V245I/G246S/F250S (“x11 green”, or “FLuc_green”). In iluc1, however, the addition of S284T alone produced a stable red-shifted variant (ilucR) almost identical in spectrum to x11 red2 (Supplementary Figure S14). The addition of S284T to a bright x11 Fluc *Ω*-loop deletion mutant (ΔP359) (Halliwell et al., 2018) also produced a bright red-shifted mutant, x11 S284T/ΔP359, and these gave 2.3 and 2.4% of activity with *DL*-iLH_2_ compared to WT Fluc with *D*-LH_2_, respectively. To blue-shift iluc1, double mutant V2451/G246S (ilucG1), single mutant F250S (ilucG2) and triple mutant V2451/G246S/F250S (ilucG3) were constructed, giving 1, 3.6, and 1.7% of activity with *DL*-iLH_2_ compared to WT Fluc with *D*-LH_2_, respectively. All these produced blue-shifted spectra compared to iluc1. CBR was the most red-shifted variant tested (peaking in the 722 nm filter) (Supplementary Figure S15) but was relatively dim compared to red-shifted ilucs with *DL*-iLH_2_ (0.2%) and, thus, not further examined.

### 2.4 Dual-cell type unmixing with infraluciferin in human embryonic kidney 293 cells

**FIGURE 7:**
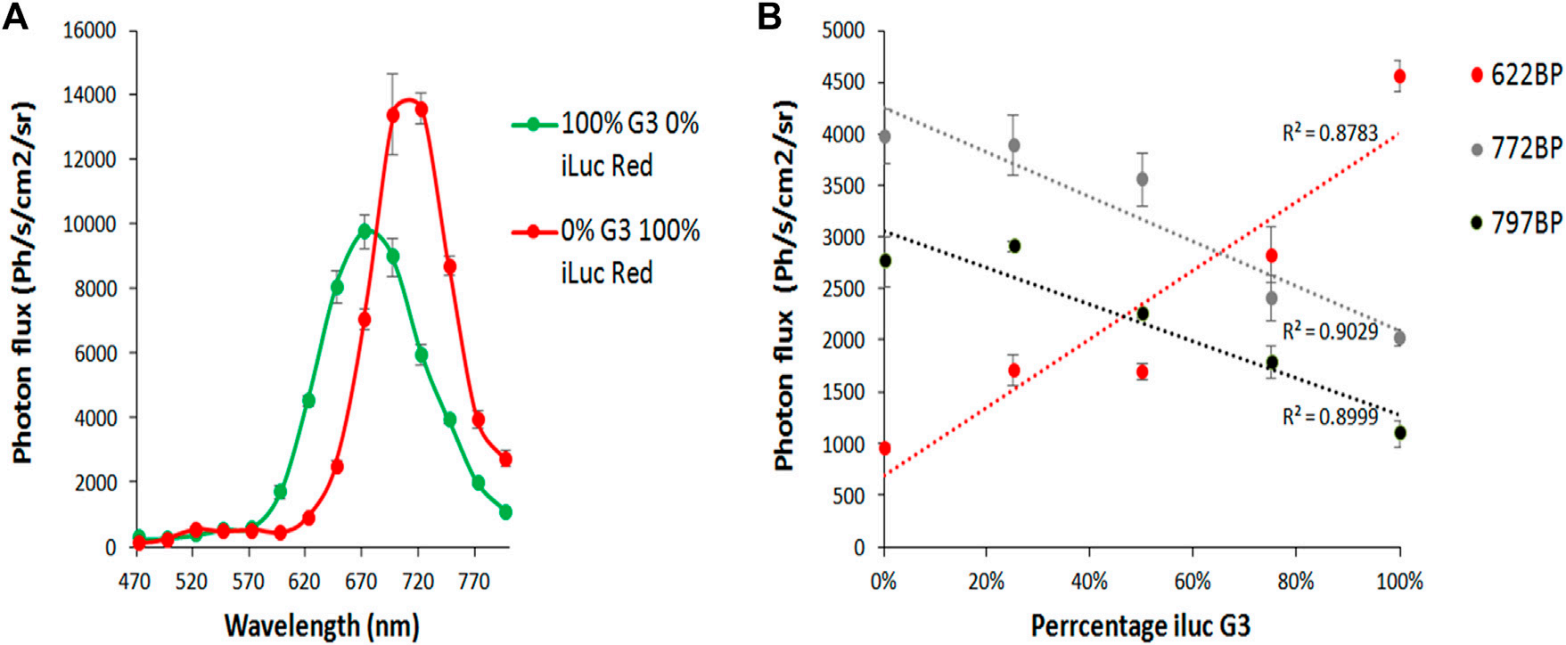
Imaging and unmixing HEK 293 cells expressing dual mixes of ilucG3 and ilucR. Substrates were dispensed with a multiwell pipette and, after 5 min, were imaged in successive filters on the PIO (622, 722, and 797 nm band-passes (BP)). Regions of interest were analyzed for photon flux at different wavelengths using the M3 Vision software. **(A)** Quantitative bioluminescence spectra from variants. **(B)** Increasing percentage of ilucG3 imaged through 622, 722 and 797BP filters.

To test the ability to simultaneously image and resolve HEK 293 cells *in vitro* expressing the different colored iluc mutants from each other with *DL*-iLH_2_, potential dual color pairings ilucG2/x11 S284T ΔP359 and ilucG3/ilucR were compared in HEK cell unmixing experiments. 1.5 × 10^6^ HEK 293 cells were plated in a 48-well format and transfected after 24 h with mixes (0, 25, 50, 75, and 100%) lentiviral vectors, encoding either of the dual far-red to nIR pairs and after 24 h were imaged by the addition of either *D*-LH_2_ or *DL*-iLH_2_. The brightest pairing, ilucG2 and x11 S284T ΔP359, was not found to be separable due to variation in an unstable spectrum of ilucG2; however, ilucG3 and ilucR spectra were stable and increasing amounts of cells (ca.7800-31,250) in mixes could be imaged in separate far-red to nIR band-pass filters in the PIO with *DL*-iLH_2_ (Figures 7A and B).

## 3 Concluding remarks

We report an unexpected finding that the bioluminescence of Eluc with *DL*-iLH_2_ is in the visible region (green) and that pairing it with other enzymes produces unprecedentedly large single-substrate bioluminescence wavelength separations. Thus, synthetic bioluminescence using *DL*-iLH_2_ has more than double the possible spectral range of natural firefly bioluminescence without the requirement for resonance energy transfer acceptors. This basic discovery could have ramifications in academia in terms of the color tuning mechanism of beetle luciferases and in bioimaging with the engineering of enzymes that are brighter at visible wavelengths, and enzymes with emission colors intermediate between green and far-red. We have identified enzymes with improved activity, good color stability, and emission kinetics with *DL*-iLH_2,_ which could be useful as first-generation infraluciferases (ilucs) for use in dual *in vivo* imaging and continue to test and engineer new generation ilucs for potential application to simultaneous single-substrate multiparametric BLI in the future.

### 3.1 Contribution to the field statement

Synthetic bioluminescence with infraluciferin has advantageous properties over naturally evolved bioluminescence for applications in bioimaging. With an extended spectral range, in the future, enzymes may be engineered with high activity emissions ranging from the green to the nIR and improve the amount of biological information that can be imaged simultaneously from living systems.

## 4 Materials and methods

### 4.1 Synthesis of luciferins and luciferin source

*D*-luciferin potassium salt was purchased from Regis Technologies (CA, United States). The free acid and methyl ester of *DL*-infraluciferin were synthesized as described within the literature (Anderson et al., 2017) and prepared by first dissolving them to 10 mg/ml in DMSO and then further in a relevant buffer.

### 4.2 Vectors, cloning, over-expression, and purification of enzymes

Vectors encoding WT, x2, x5, and x11 Flucs in pET16b were prepared in previous work. 10X-N-terminal His-tagged Eluc and CBR were amplified from pLR6-Eluc (GenBank KU756582.1), provided kindly by Mikhail Koksharov (Brown University, United States), or pGex-CBR plasmids, respectively, and cloned into the pET16b with NcoI and BamHI. These were transformed into *E. coli* BL21 (DE3) pLysS cells (Agilent Technologies, CA, United States) and over-expressed and purified by nickel-NTA affinity chromatography as described previously (Law et al., 2006; Jathoul et al., 2012a). The pCCL vector 305 was kindly provided by Prof. Riccardo Brambilla (Cardiff University, Cardiff, United Kingdom) under MTA.

### 4.3 Construction of random mutants at positions E354 and D357 by overlap extension mutagenesis

Overlap extension was used to introduce mutations into Flucs by site-directed random mutagenesis since this method gives added versatility when introducing simultaneous mutations at different sites. To construct random mutations at E354 and D357, first, a fragment of ca.1070 bp was amplified from pET16b x2 Fluc using outer primer pETPpyFor (AGG​TCG​TCA​TAT​GGA​AGA​CGC​CAA​AA) and overlap primer RYrRev (GACCGCGCCCGGTTTNNNATCCCCNNNGGGTGTAATCAGAATAG). Another ca.580 bp fragment was amplified separately amplified using overlap primer RYrFor (CTATTCTGATTACACCCNNNGGGGATNNNAAACCGGGCGCGGTC) and outer primer pETPpyRev (GCA​GCC​GGA​TCC​AGT​TAC​ATT​TTA​CA). Each fragment was gel purified, and they were fused together by PCR using outer primers pETPpyFor and Rev to produce a single band of ca.1500bp, which was further extracted and digested with NdeI and BamHI before subcloning into pET16b. Luminescent colonies were identified by spraying with 60 μM DL-iLH_2_ in 0.1M citrate buffer (pH 5) and being imaged for 1 min in the PIO and were verified by sequencing.

### 4.4 Measurement of specific activity, coenzyme A assays, and flash kinetics

For specific activity, each enzyme was prepared in chilled PEM (1x PBS, 2 mM ethylenediaminetetraacetic acid (EDTA) and 10 mM magnesium sulphate (MgSO_4_), pH 7.3) or TEM buffer (100 mM Tris-acetate, 2 mM ethylenediaminetetraacetic acid (EDTA) and 10 mM magnesium sulphate (MgSO_4_), pH 7.8) and 200 μM *D*-LH_2_ or 60 μM *DL*-iLH_2_ and 2 mM ATP at pH 7.8 were used to saturate reactions. Measurements were captured with or without band-pass filters in the PIO 1 min after addition of substrates, for periods of up to 1 h at 28–30°C. Flash kinetics were measured with both substrates by dispensing 2 mM ATP onto 0.167 μM enzymes with 200 μM *D*-LH_2_ or 60 mM iLH_2_ and adjusting PMT gain values to 1,500 v for *D*-LH_2_ and 4,095 v for *DL*-iLH_2_. To test the effect of coenzyme A (CoA), 200 μM was also supplemented into experiments. To construct the Hb phantom, 50% whole equine blood in 1% agarose was allowed to set in a 150 mm square Petri dish, and 96-well plates containing samples were overlaid with this to test the effect of filtering of emission by blood.

### 4.5 pH dependence of bioluminescence spectra

Bioluminescent spectra were captured using both the PIO and Clariostar multimeter (BMG Labtech, Ortenburg, Germany). For measurements at differing pH values, TEM buffer was adjusted to different pH values using acetic acid or sodium hydroxide, and these were used to prepare both substrates and enzymes.

### 4.6 Kinetics assays: measurement of K_m_ for ATP

For ATP K_m_ measurements, light emission was measured when substrate concentrations of ATP were varied between 1 μM and 2 mM in the presence of either saturating *D*-LH_2_ or *DL*-iLH_2_, respectively. Data were plotted using the Hanes–Woolf plot to derive K_m_ and k_cat_ values (obtained with a BMG Fluostar PMT gain of 4,095 v).

### 4.7 Chemiluminescence of infraluciferin Me ester

To initiate chemiluminescence, 50 μl of 10 mg/ml iLH_2_ Me ester was added to 200 μl 2M t-BuOK in TEM buffer or 5 μl of ester was added to 50 μl 1M t-BuOK in DMSO and light emission was integrated over 1 min using different band pass filters in the PIO.

### 4.8 HEK cell transfection, transduction, and imaging

HEK cells were plated at the density of 1.5 × 10^6^ cells per plate in a 6-well dish. The following day, wells were transfected with 500 ng pCCL vectors encoding Flucs using 3 μl of GeneJuice transfection reagent (Novagen, WI, United States) and imaged after 48h for EGFP expression using an excitation wavelength of 487 nm and acquisition with a 547 nm (+/−15 nm) filter, and subtraction of the subsequent image taken with an excitation value of 412 nm. BLI was then acquired for 5 min after dispensing substrates on cells using a multiwell pipette.

## Data Availability

The original contributions presented in the study are included in the article/Supplementary Material; further inquiries can be directed to the corresponding author. Data and materials will be made available in line with UKRI policies.
