# Supplementary material for: A higher spectral range of beetle bioluminescence with infraluciferin
Source: Front Bioeng Biotechnol. 2022 Aug 26;10:897272. doi: 10.3389/fbioe.2022.897272 (PMC9459109; doi:10.3389/fbioe.2022.897272)
Supplement: Supplementary file 1 [file DataSheet1.pdf]

Supplementary Material

1 Supplementary Data

1.1 Supplementary Figures

a.

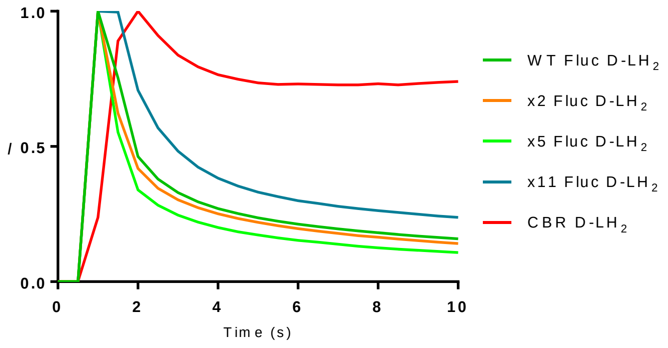

b.

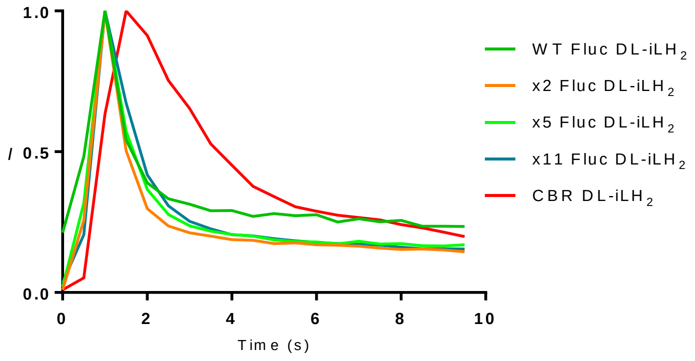

c.

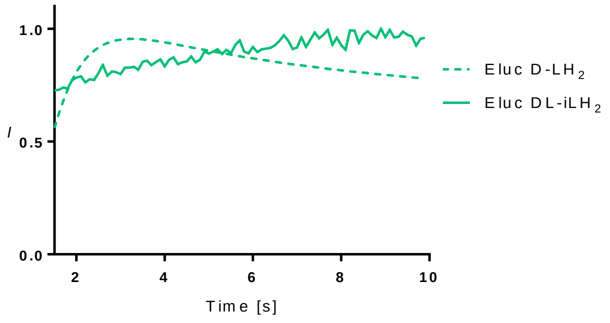

Supplementary Figure 1. Normalised flash kinetics of enzymes with *D*-LH_2 and *DL*-iLH_2. 2mM ATP was injected into reactions containing 500μM *D*-LH_2 (a) or 50μM *DL*-iLH_2 (b) and 0.167μM luciferases and emission kinetics were captured in a Fluostar Optima luminometer (BMG Labtech, Ortenburg, Germany). In (c) flash kinetics of Eluc were captured using a BMG Clariostar.

**Enzyme**	**Km for ATP_{iLH2} (μM)**	**Kcat (RLU s^{-1}) ($x10^{16}$)**
WT	160	0.8
x2	200	4
x5	350	2
x11	200	4
Eluc*i*	200	ND
CBR	350	1

Supplementary Table 1. Km values of enzymes for Km for ATP in the presence of iLH_2 ($ATP_{(DL\text{-}iLH2)}$). Reactions were initiated by addition of 200 μM *DL*-iLH_2 to different concentrations of ATP (c.1-2000 μM at pH 7.8) with 25 pmol enzymes and light emission was measured using a

BMG Fluostar luminometer over 10 s at a PMT gain value of 4095 v. [i]The Km of Eluc for ATP$_{(DL\text{-}iLH2)}$ was determined separately in the Clariostar instrument, using x11 Fluc as a control.

a. **b.**

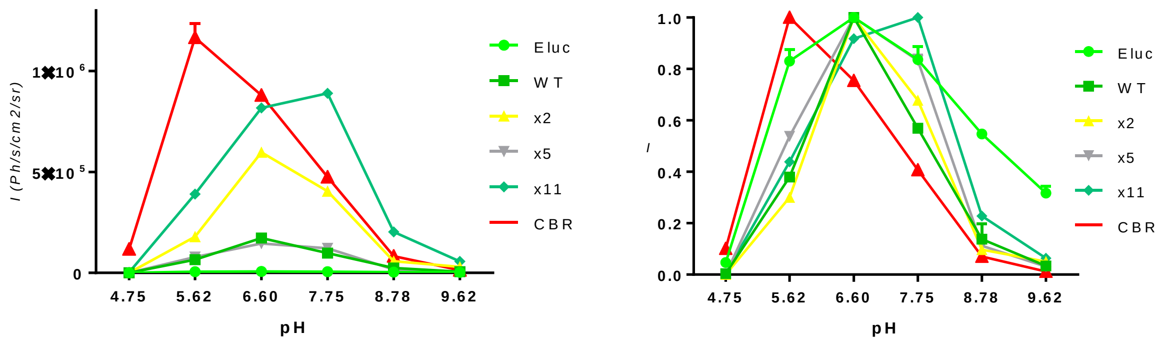

Supplementary Figure 2. **pH dependence of activity of luciferases with *DL*-iLH$_2$.** a. 0.1μM enzymes assayed with 60μM *DL*-iLH$_2$ and 2mM ATP at different pH values by addition of acetic acid or NaOH to TEM buffer. b. Normalised values.

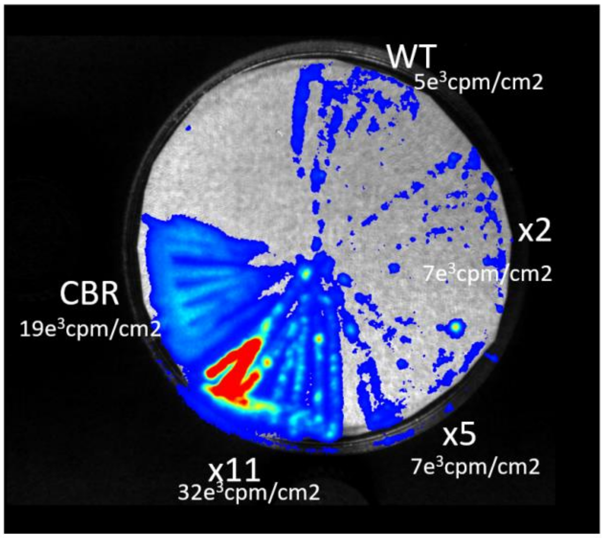

Supplementary Figure 3. Picture of *E. coli* BL21 (DE3) plysS expressing *Ppy*-based Flucs and CBR. Colonies from a petri dish were lifted onto a nitrocellulose membrane induced on 1mM isopropyl β-D-1-thiogalactopyranoside (IPTG) for 3 hrs at room temperature and sprayed with 60µM iLH2 in 0.1M citrate buffer (pH5) before imaging for 30s with an open filter in the PIO.

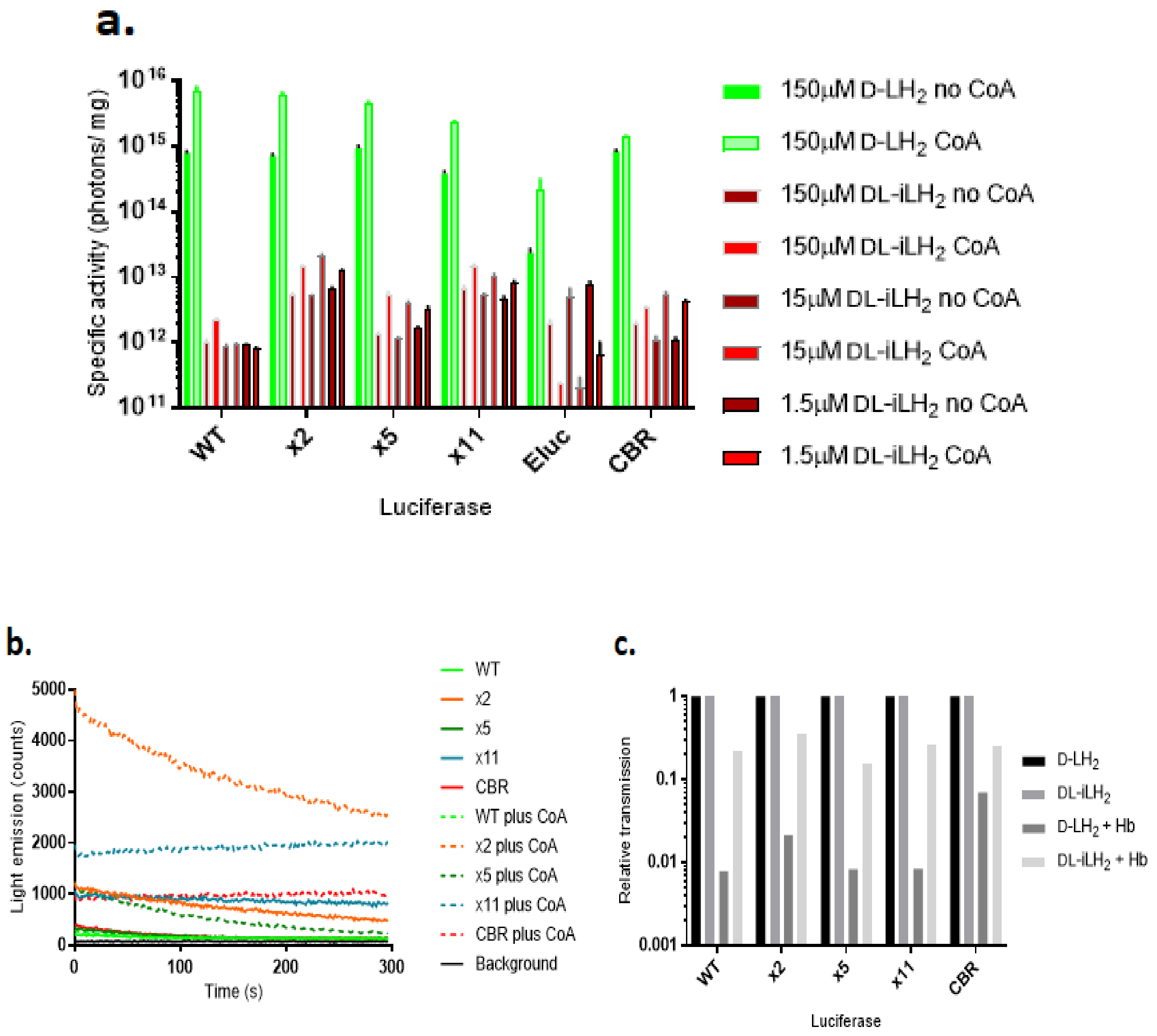

Supplementary Figure 4. Effect of substrate concentration, coenzyme A and haemoglobin on light levels with *DL*-iLH$_2$. a. Specific activity of enzymes with CoA at pH 7.8. P-values from t-tests comparing enzyme activities with *DL*-iLH$_2$ with WT Fluc. Without CoA x2, x5, x11 and CBR P-values are <0.0001, <0.0001, 0.0001 and 0.0169, respectively. With CoA x2, x5, x11 and CBR P-values are <0.0001, <0.0001, 0.0001 and 0.0169, respectively. b.i. Light yield of WT Fluc, x2, x5, x11 and CBR with 150μM *D*-LH$_2$ or 50μM *DL*-iLH$_2$ and 2mM ATP in 2 mM EDTA and 10 mM magnesium sulphate in PBS at pH 7.4. Results of t-tests are shown comparing activity of enzymes with *DL*-iLH$_2$ with WT Fluc. P-values for x2, x5, x11 and CBR are <0.0001, <0.0001, 0.0001 and <0.0001, respectively. b. Emission kinetics with *DL*-iLH$_2$ with CoA. Eluc omitted. c. Histogram showing the relative penetration of emission from different substrates through the blood phantom. Green emitting Eluc omitted.

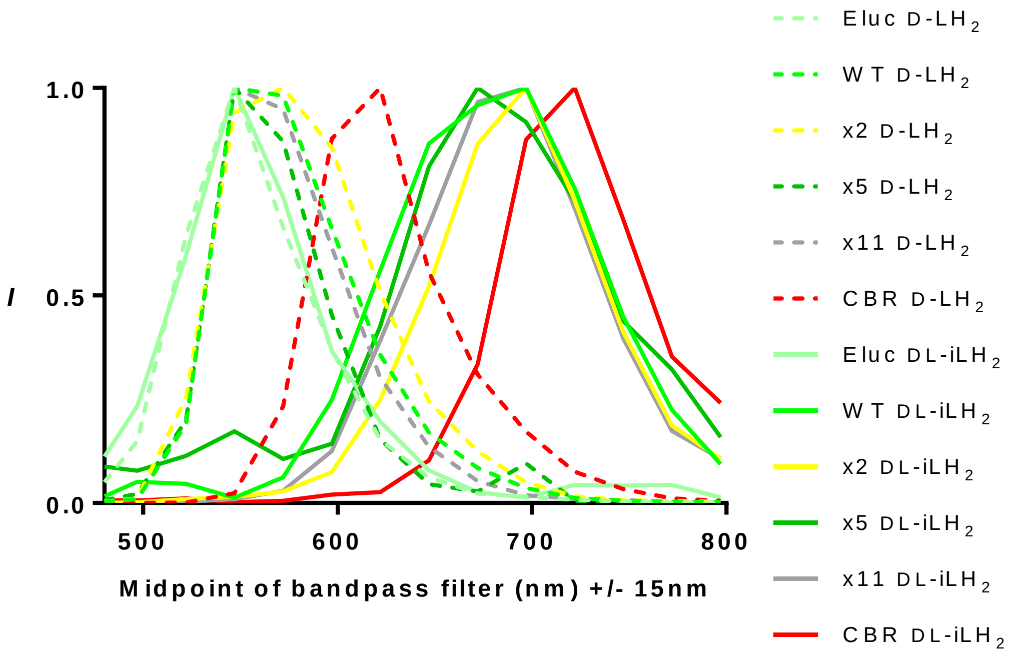

Supplementary Figure 5. Normalised bioluminescence spectra of 0.5μM enzymes acquired in the PhotonIMAGER Optima small animal imager. 200μM *D*-LH$_2$ and 20μM *DL*-iLH$_2$ applied to

0.5μM enzymes with 2mM ATP and light emission captured for 20s - 1 min through different band-pass filters (15nm pass-bands).

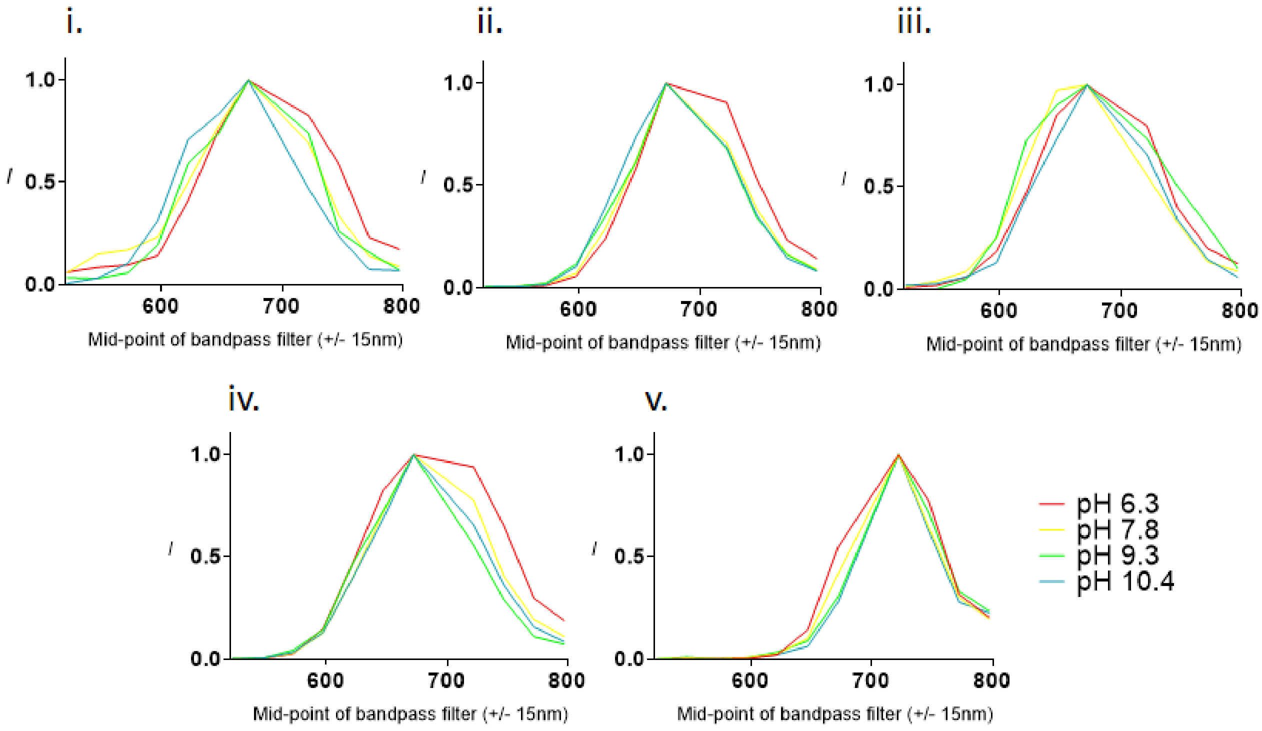

Supplementary Figure 6. **pH dependence of bioluminescence spectra of luciferases** (i-v: WT, x2, x5, x11 and CBR) with *DL*-iLH$_2$ showing bathochromic shifts at low pH. See Materials and Methods for details.

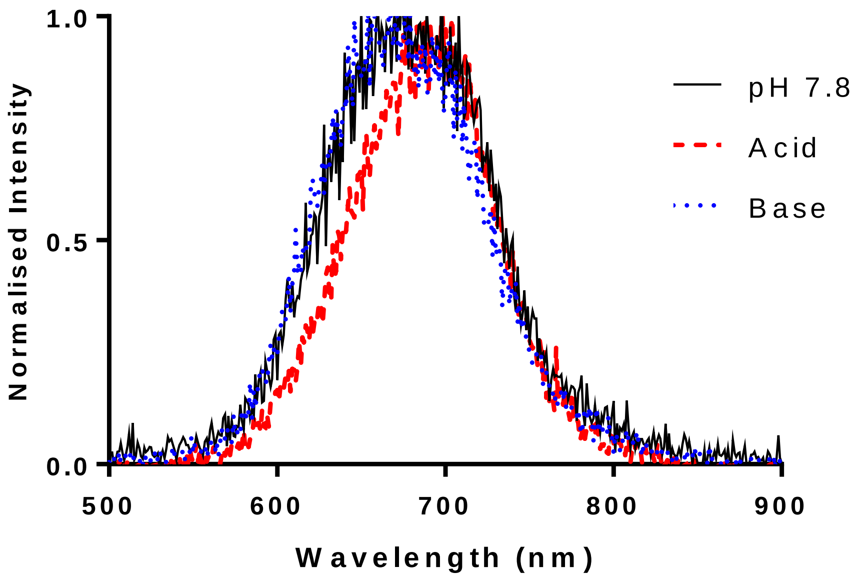

Supplementary Figure 7. **Bathochromic shift of WT Fluc with *DL*-iLH2.** Details as in Figure 6, however, 5ul 5M NaOH or 2ul glacial acetic acid was added where indicated. Spectrum measured in a Varian Cary Eclipse spectrofluorometer (Varian, CA, USA). Data not corrected for photomultiplier spectral sensitivity.

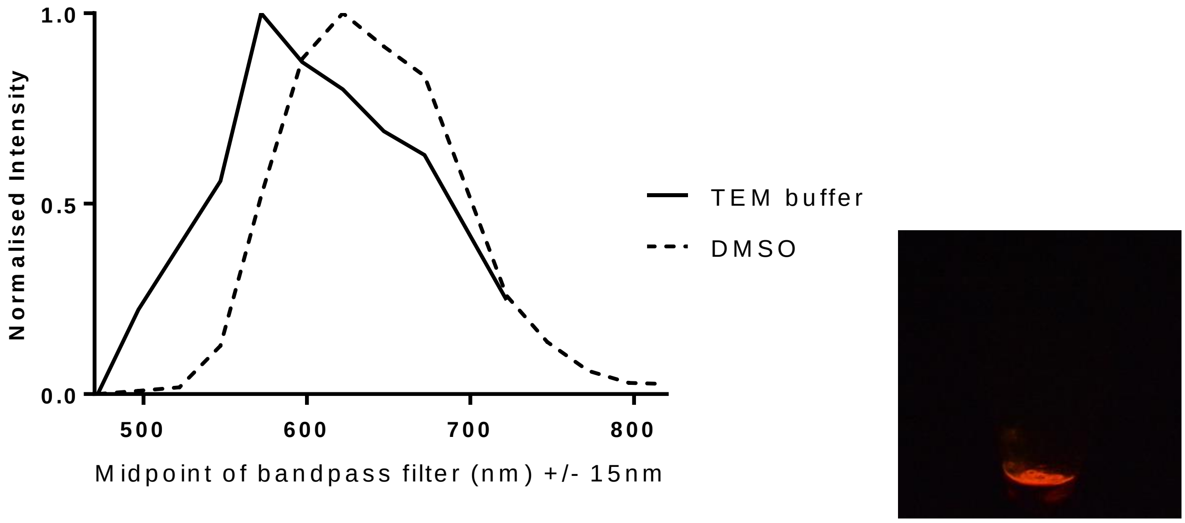

Supplementary Figure 8. Chemiluminescence of *DL*-iLH$_2$ methyl ester in tris, EDTA and magnesium sulphate buffer (TEM buffer) and dimethyl sulphoxide. To initiate chemiluminescence 50μl of 10mg/ml iLH$_2$ ester was added to 2M potassium tert-butoxide (t-BuOK) (Miura et al., 2013) in TEM buffer or5μl of 10mg/ml iLH$_2$ ester was added to 1M t-BuOK in DMSO and emission was captured in the PIO in different bandpass filters. Chemiluminescence of *DL*-iLH$_2$ Me ester displayed green color in buffer (peak in 547nm band-pass filter of the PIO) and a slightly brighter red color (peak in 622nm filter) in dimethyl sulfoxide. Therefore, chemiluminescence colors of *DL*-iLH$_2$ ester are akin to those of *D*-LH$_2$, relatively blue-shifted compared to the bioluminescence emission colors generated with majority of enzymes. Inset image (bottom right): DSLR camera photograph of chemiluminescence of *DL*-iLH$_2$ ester in DMSO.

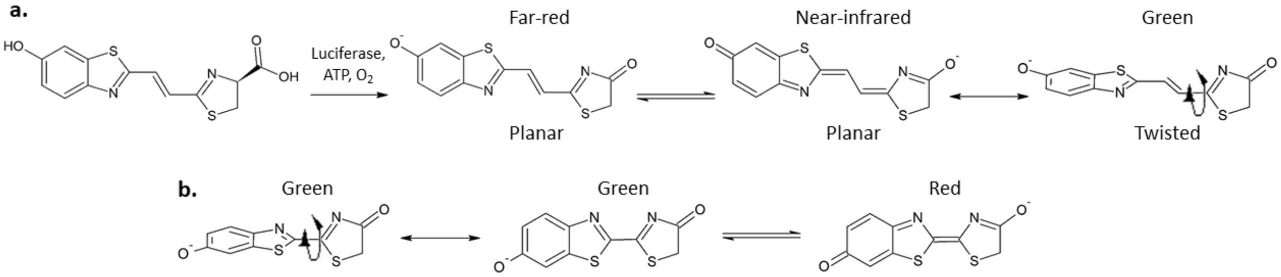

Supplementary Figure 9. a. Proposal for extension of the 'specific' structural mechanism of color regulation with infraluciferin in luciferase. This is based on the mechanisms with natural luciferin by Branchini et al., 2004[1] and 2017[2], observations with infraluciferin and those reported with other analogues[3]. b. Potential analogous equilibrium of natural oxyluciferin (LO) conformations and associated colors. In both cases planarity could be stabilised by pi-stacking of benzothiazole moieties with Phe 247 in *Ppy* Fluc. Green chemiluminescence has been observed with red-shifted

bioluminescent biphenyl luciferin analogues, and rotation of LO forms in solution was proposed as an explanation[3]. One proposal for the structure of the emitters of LO is an equilibrium between two separately conjugated resonance forms[1, 2], stabilised by different active site hydrogen bonding between R218, N229, Y255, S284, E311, R337 and a central water molecule in the active site proximal to the 6'-hydroxyl of substrates. Such a 'specific'[4] mechanism could be envisaged with infraoxyluciferin (iLO), but with two additional central carbon atoms and two extra rotatable bonds, iLO has more degrees of freedom than LO. Rotation at the central axis of iLO could limit conjugation between benzothiazole (BT) and thiazolone moieties and limit electron delocalisation across the alkene linker, resulting in the blue-shifts observed. In such a scenario it would be possible to engineer Fluc color mutants spanning from green to nIR with iLH_2 by stabilising different (at each linker carbon) twisted or planar conformations of excited state iLO. We suggest that the green spectrum of Eluc with iLH_2 may be controlled by rotation of excited state iLO in the active site of Eluc. We attempted random mutagenesis of Eluc at position F243 (equivalent to F247 in *Ppy* Fluc) and although we observed blue-shifting in *E. coli* colonies with Eluc F243L, this effect was not carried through to pure protein of the mutant, which emitted green light similar to Eluc but significantly reduced in activity.

a. **b.** **c.**

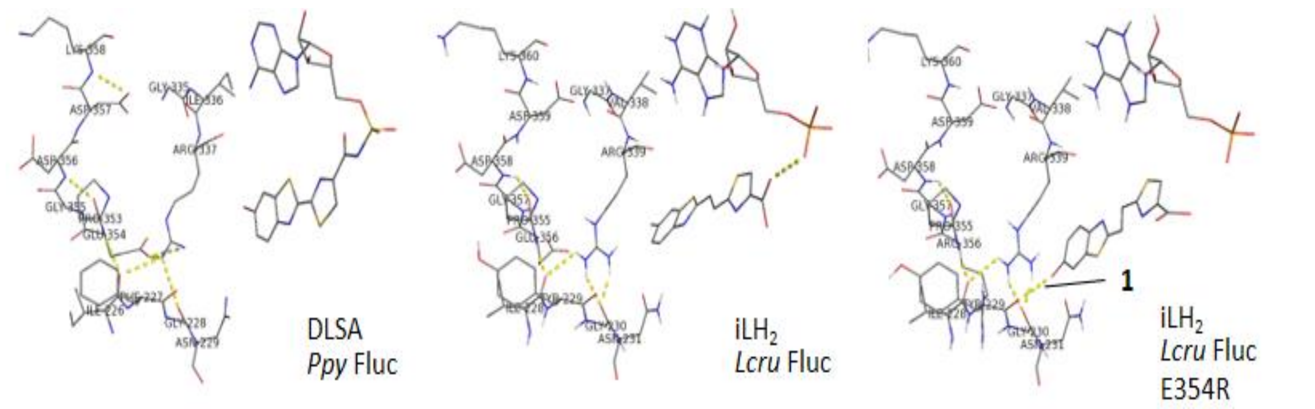

Supplementary Figure 10. **a.** Structure of DLSA complex and region of E354 and D357 displayed in *Ppy* Fluc structure 4G36.pdb[5]. **b.** Docking of *D*-iLH_2 into AMP bound Fluc structure from *L. cruciata* (*Lcru*) luciferase 2D1Q.pdb[6]. **c.** Docking of *D*-iLH_2 into AMP bound Fluc structure from *Lcru* 2D1Q.pdb containing *in silico* energy minimised mutation E356R (equivalent to *Ppy* Fluc E354R) showing additional H-bond (labelled **'1' in c**). We performed *in silico* docking of *D*-iLH_2 into the *Lcru* Fluc crystal structure with AMP bound (2D1Q.pdb) and a 2D1Q.pdb E356R mutant (equivalent to *Ppy* Fluc E354R) we generated. *D*-iLH_2 docked into the active site of WT *Lcru* Fluc superimposing well with position of luciferin in the DLSA complex of 4G36.pdb, binding primarily as a cis-isomer with twisting between the benzothiazole (BT) and thiazoline moieties with potential for binding modes with free rotation of the BT with respect to the thiazoline. Figure prepared using Pymol (Molecular graphics system, version 2.0, Schrodinger, LLC) and AutoDock Vina[7]. Mutation and energy minimisation carried out using the GROMACS plugin in Swisspdviewer[8].

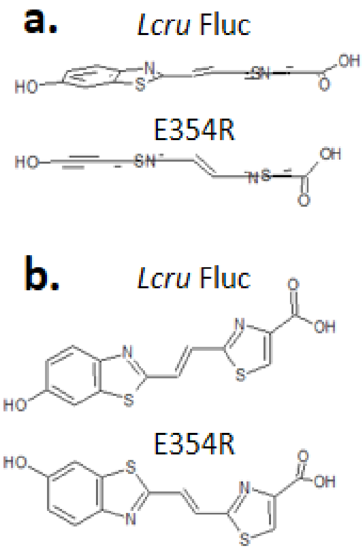

Supplementary Figure 11. Conformations of iLH_2 generated during docking into *Lcru* and mutant, extracted from pdb files of docking analyses and displayed using Chemsketch (ACD/Labs Inc., Ontario, Canada). a. Side-view of main binding modes. b. Top-down view of main binding modes. *D*-iLH_2 docked into the *Lcru* E356R structure (lower image in b) as trans-isomer with an additional hydrogen bond between the main chain carbonyl of Y229 and the hydroxyl of *D*-iLH_2, fixing the molecule relatively planar in the active site, congruent with the calculated likely conformation of emitter of iLO (‘OxyiLH_2-2’)[9], suggesting E354R may allow better coordination of iLH_2 in the active site prior to oxidation.

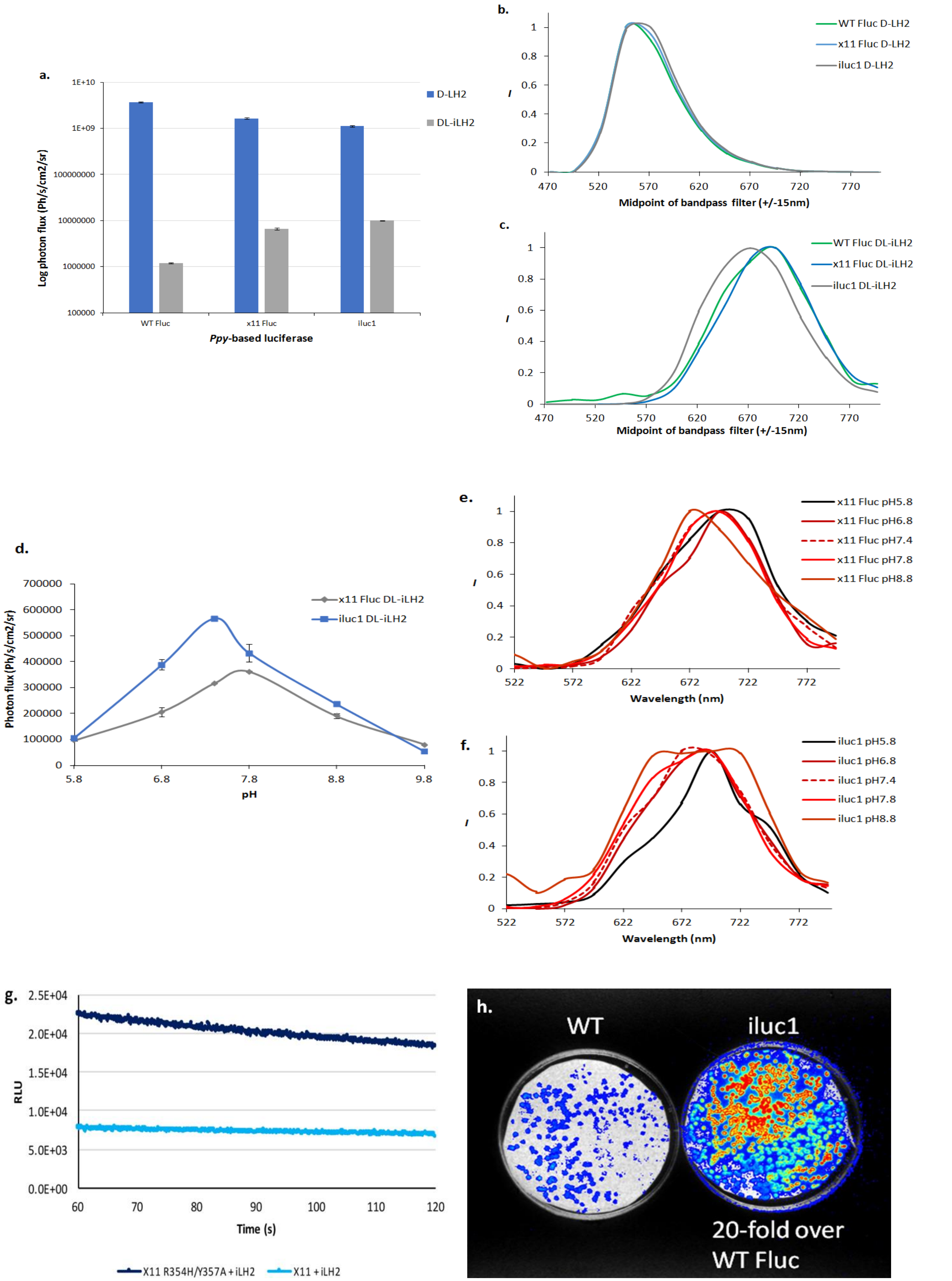

a.
D-LH2
DL-iLH2
Log photon flux (Ph/s/cm2/sr)
1E+10
1E+09
100000000
10000000
1000000
100000
WT Fluc
x11 Fluc
iluc1
Ppy-based luciferase
b.
WT Fluc D-LH2
x11 Fluc D-LH2
iluc1 D-LH2
I
Midpoint of bandpass filter (+/-15nm)
c.
WT Fluc DL-iLH2
x11 Fluc DL-iLH2
iluc1 DL-iLH2
I
Midpoint of bandpass filter (+/-15nm)
d.
x11 Fluc DL-iLH2
iluc1 DL-iLH2
Photon flux (Ph/s/cm2/sr)
pH
e.
x11 Fluc pH5.8
x11 Fluc pH6.8
x11 Fluc pH7.4
x11 Fluc pH7.8
x11 Fluc pH8.8
I
Wavelength (nm)
f.
iluc1 pH5.8
iluc1 pH6.8
iluc1 pH7.4
iluc1 pH7.8
iluc1 pH8.8
I
Wavelength (nm)
g.
RLU
Time (s)
X11 R354H/Y357A + iLH2
X11 + iLH2
h.
WT
iluc1
20-fold over WT Fluc

Supplementary Figure 12. Specific activities (**a.**) and bioluminescence spectra 200μM *D*-LH_2 (**b)** or *DL*-iLH_2 (**c.**) 0.167μM of purified WT, x11 Fluc and iluc1 with, and 2mM saturating ATP at pH 7.4. pH dependence of activity (**d.**) and bioluminescence spectra with *D*-LH_2 (**e.)** or *DL*-iLH_2 (**f.**) also at these concentrations, but other conditions as in Supplementary Figure 2. Kinetics of x11 Fluc and iluc1 with *DL*-iLH_2 (**g.**) captured over 2 min, 1 min after the initiation of bioluminescence. Reactions in 96 well plates contained 0.05 mL of 0.1 mM of the indicated analog and 5 μL of 0.05 mg/ml enzyme in 50 mM Tricine pH 7.8. Image (**h.**) of *E. coli* BL21 expressing WT *Ppy* Fluc (left) and iluc1 (right) on nylon membranes lifted from petri dishes and imaged as in Supplementary Figure 3.

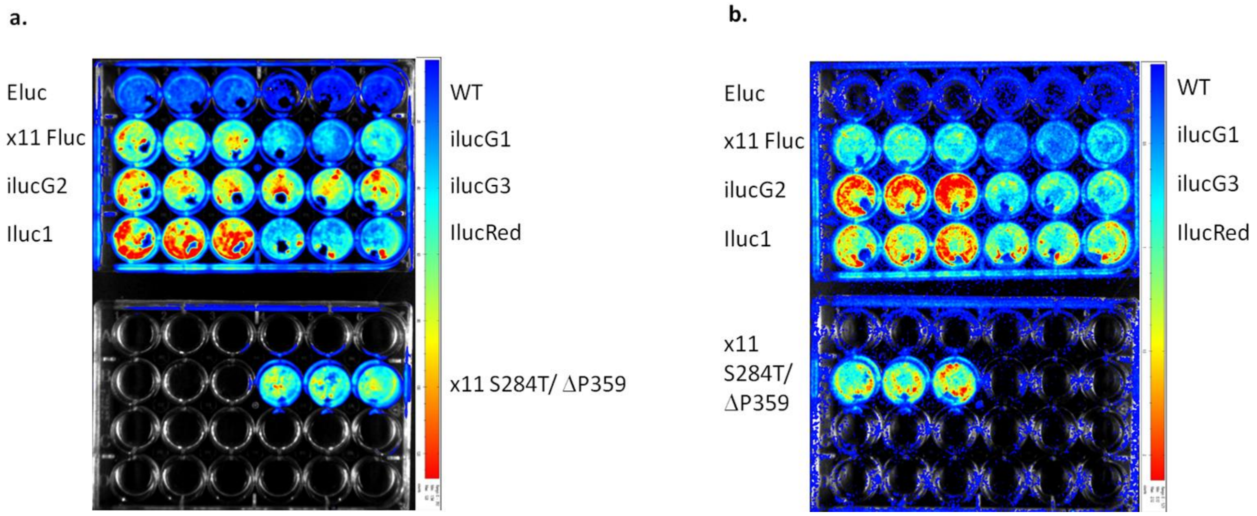

Supplementary Figure 13. Open filter images of HEK 293 cells expressing different luciferases captured in the PIO. Details as in Figure 5 of the main text. a. *D*-LH_2 K^+ salt, b. *DL*-iLH_2 Me ester.

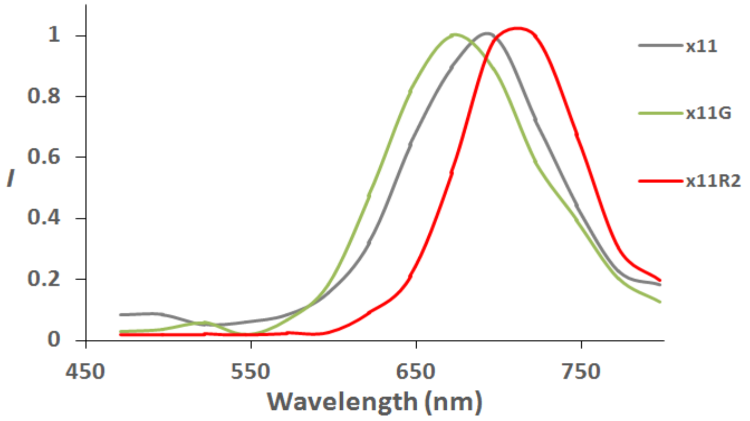

Supplementary Figure 14. Bioluminescence spectra of dual color variants used in Stowe et al., 2019 for dual parameter imaging in mouse models of cancer *in vivo:* x11G (x11 V2451/ G246S/ F250S) is ‘FLuc_green’ and x11R2 (x11 S284T R354I) is ‘FLuc_red’ in that paper.

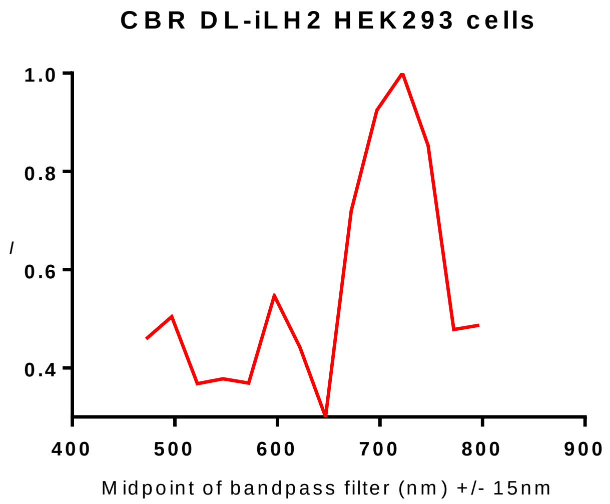

Supplementary Figure 15. Weak bioluminescence spectrum of CBR with *DL*-iLH_2 in HEK cells acquired in the PIO by imaging in different band-pass filters.

a.

	100%	75%	50%	25%	0%				
A + B	100% A	75% A	50% A	25% A	0% A				
A + B	100% A	75% A	50% A	25% A	0% A	0% C	0% C	0% C	0%
A + B	100% A	75% A	50% A	25% A	0% A	25% C	25% C	25% C	25%
B + C	100% B	75% B	50% B	25% B	0% B	50% C	50% C	50% C	50%
B + C	100% B	75% B	50% B	25% B	0% B	75% C	75% C	75% C	75%
B + C	100% B	75% B	50% B	25% B	0% B	100% C	100% C	100% C	100%
						C+ A	C+ A	C+ A	

b.

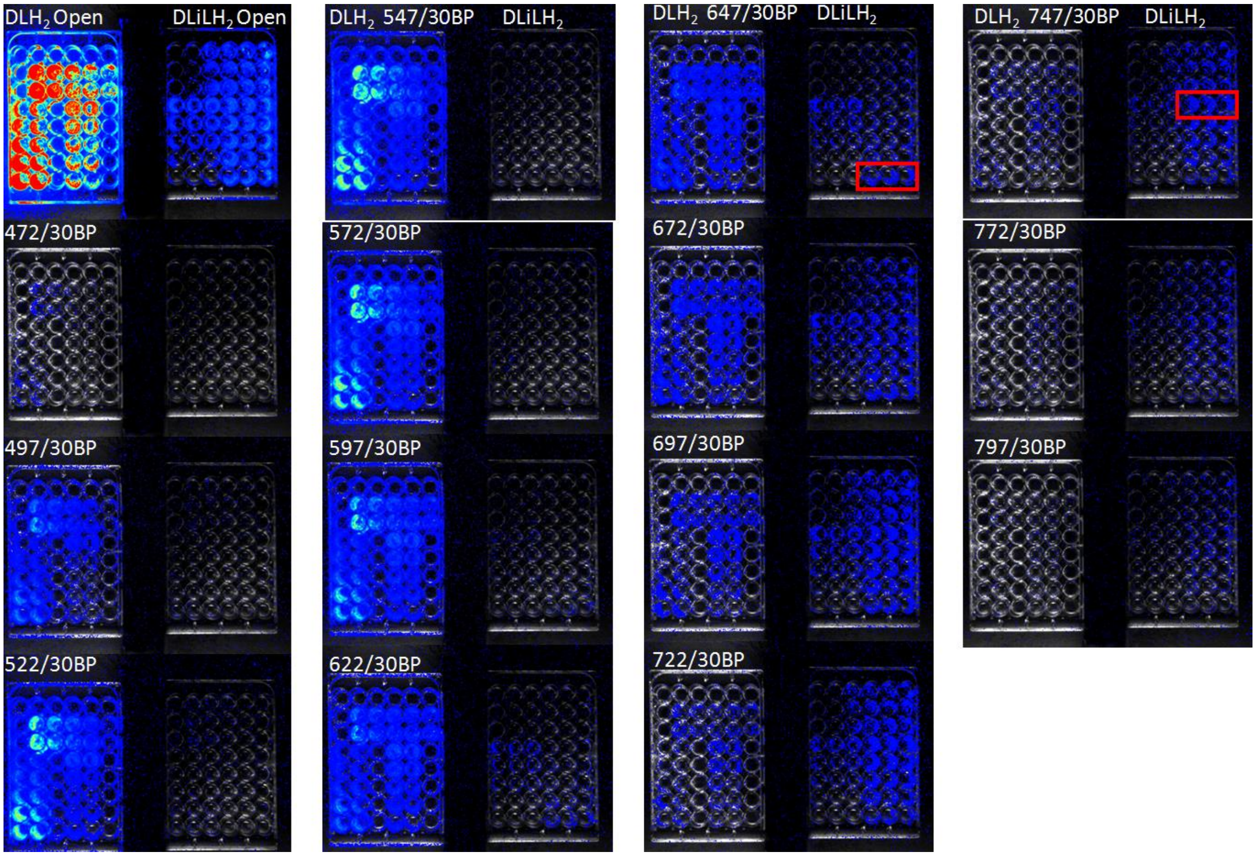

Supplementary Fig. 16. a. Set-up for transfection mixes of lentiviral vectors encoding luciferases for experiments in a 48-well format (A: Eluc, B: ilucG3 and C: ilucR). **b.** BLI of Eluc, ilucG3 and ilucR in mixes imaged in Figure 6 of the main text, captured in separate band-pass filters in the PIO with *D*-LH_2 and *DL*-iLH_2 at the same LUT setting.

Supplementary references

[1] Branchini BR, Southworth TL, Murtiashaw MH, Magyar RA, Gonzalez SA, Ruggiero MC and Stroh JG (2004). *An alternative mechanism of bioluminescence color determination in firefly luciferase. Biochemistry.*43:7255-62.

[2] Branchini BR, Southworth TL, Fontaine DM, Murtiashaw MH, McGurk A, Talukder MH, Qureshi R, Yetil D, Sundlov JA and Gulick AM (2017). Cloning of the Orange Light-Producing Luciferase from *Photinus scintillans* - A New Proposal on how Bioluminescence Color is Determined. *Photochem. Photobiol.* 93, 479-485.

[3] Miura C, Kiyama M, Iwano S, Ito K, Obata R, Hirano T, Maki S and Niwa H (2013). Synthesis and luminescence properties of biphenyl-type firefly luciferin analogs with a new, near-infrared light-emitting bioluminophore. *Tetrahedron* 69: 9726-34.

[4] Ugarova NN and Brovko LY (2002). Protein structure and bioluminescent spectra for firefly bioluminescence. *Luminescence* 17: 321-30.

[5] Sundlov JA, Fontaine DM, Southworth TL, Branchini BR and Gulick AM (2012). Crystal structure in a second catalytic conformation supports a domain alternation mechanism. *Biochemistry* 51: 6493-5.

[6] Nakatsu T, Ichiyama S, Hiratake J, Saldanha A, Kobashi N, Sakata K and Kato H (2006). Structural basis for the spectral difference in luciferase bioluminescence. *Nature Lets.* 400: 372-6.

[7] Goodsell DS and Olson AJ (1990). Automated docking of substrates to proteins by simulated annealing. *Proteins: Structure, Function and Genetics* 8: 195-202.

[8] Guex N and Peitsch MC (1997). SWISS-MODEL and the Swiss-PdbViewer: an environment for comparative protein modelling. *Electrophoresis* 18: 2714-23.

[9] Berraud-Pache R and Navizet I (2016). QM/MM calculations on a newly synthesised oxyluciferin substrate: new insights into the conformational effect. *Phys. Chem. Chem. Phys.* 18: 27460-7.
